# Modular evolution of glutathione peroxidase genes in association with different biochemical properties of their encoded proteins in invertebrate animals

**DOI:** 10.1186/1471-2148-9-72

**Published:** 2009-04-06

**Authors:** Young-An Bae, Guo-Bin Cai, Seon-Hee Kim, Young-Gun Zo, Yoon Kong

**Affiliations:** 1 Department of Molecular Parasitology, Sungkyunkwan University School of Medicine and Center for Molecular Medicine, Samsung Biomedical Research Institute, Suwon, Gyeonggi-do 440-746, Korea; 2 Department of Parasitology, School of Medicine, Wuhan University, Wuhan, PR China; 3 Department of Environmental Science, Kangwon National University, Chuncheon, Korea

## Abstract

**Background:**

Phospholipid hydroperoxide glutathione peroxidases (PHGPx), the most abundant isoforms of GPx families, interfere directly with hydroperoxidation of lipids. Biochemical properties of these proteins vary along with their donor organisms, which has complicated the phylogenetic classification of diverse PHGPx-like proteins. Despite efforts for comprehensive analyses, the evolutionary aspects of GPx genes in invertebrates remain largely unknown.

**Results:**

We isolated GPx homologs via *in silico *screening of genomic and/or expressed sequence tag databases of eukaryotic organisms including protostomian species. Genes showing strong similarity to the mammalian PHGPx genes were commonly found in all genomes examined. GPx3- and GPx7-like genes were additionally detected from nematodes and platyhelminths, respectively. The overall distribution of the PHGPx-like proteins with different biochemical properties was biased across taxa; selenium- and glutathione (GSH)-dependent proteins were exclusively detected in platyhelminth and deuterostomian species, whereas selenium-independent and thioredoxin (Trx)-dependent enzymes were isolated in the other taxa. In comparison of genomic organization, the GSH-dependent PHGPx genes showed a conserved architectural pattern, while their Trx-dependent counterparts displayed complex exon-intron structures. A codon for the resolving Cys engaged in reductant binding was found to be substituted in a series of genes. Selection pressure to maintain the selenocysteine codon in GSH-dependent genes also appeared to be relaxed during their evolution. With the dichotomized fashion in genomic organizations, a highly polytomic topology of their phylogenetic trees implied that the GPx genes have multiple evolutionary intermediate forms.

**Conclusion:**

Comparative analysis of invertebrate GPx genes provides informative evidence to support the modular pathways of GPx evolution, which have been accompanied with sporadic expansion/deletion and exon-intron remodeling. The differentiated enzymatic properties might be acquired by the evolutionary relaxation of selection pressure and/or biochemical adaptation to the acting environments. Our present study would be beneficial to get detailed insights into the complex GPx evolution, and to understand the molecular basis of the specialized physiological implications of this antioxidant system in their respective donor organisms.

## Background

Reactive oxygen species (ROS) are generated through an incomplete reduction of oxygen molecules during mitochondrial respiration and/or cytosolic metabolism. Exposure to exogenous stimuli such as radiation and redox-cycling drugs might be an alternative pathway of ROS production. ROS perform physiological roles relevant to cell signaling and redox-status control [1,2], while unbalanced generation of these species induces detrimental oxidation of macromolecules including DNA, proteins, and lipids. To minimize ROS-derived damage, aerobic organisms have evolved a series of multi-layered enzymatic and non-enzymatic defense systems [3]. Distinct enzymatic activities such as catalase, glutathione peroxidase (GPx), and peroxiredoxin (PRx; also called thioredoxin peroxidase) have been well characterized from numerous taxa, as the major antioxidant defense mechanism.

Selenium-containing GPx proteins reduce H_2_O_2 _and organic hydroperoxides by employing glutathione (GSH) as an electron donor. A total of eight GPx families have been described in mammals on the basis of primary structure, specific substrate accessibility, and spatial expression [4,5]. These homotetrameric isoenzymes conserve structural/biochemical properties, however, a number of enzymes that have been classified into GPx4 (phospholipid hydroperoxide GPx; PHGPx) may function in monomeric forms and exhibit unique substrate availability. The enzymes can interfere directly with hydroperoxidized phospholipids in biomembranes. Proteins belonging to the other GPx families display substrate preference toward H_2_O_2_and protect against lipid peroxidation via a concerted operation with phospholipase [6]. PHGPx is the basis of a principal defense system that intimately participates in the repair of disrupted biomembranes [7]. The vertebrate-specific GPx7 and GPx8 also lack the oligomerization loop, although their unique enzymatic properties are less understood [5].

Multiple isoenzymes showing primary structure similar to those of the mammalian PHGPxs have been described in plants, along with their respective subcellular expression profiles [8,9]. Plant enzymes possess a Cys residue instead of a selenocysteine (Sec) at the catalytic site, and prefer thioredoxin (Trx) as the electron source [9-11]. A pair of PHGPx-like proteins that effectively reduce the peroxides by adapting the Trx system has also been isolated from insect, yeast, and protozoa [12-15]. Interestingly, the green alga *Chlamydomonas reinhardtii *was likely to express both GHS-dependent (CrGPx1 and CrGPx2) and Trx-dependent (CrGPx3–5) GPxs [16]. These observations have created a controversy regarding the classification of PHGPx-like proteins [8,9]. Conventional cladistic analyses based on comparison of primary structures generally annotate these proteins as PHGPxs, prior to empirical examination of their catalytic mechanisms (for example, see [17]). It has been suggested that the Trx-dependent GPxs comprise the fifth class of the PRx family, on the basis of their biochemical properties rather than their phylogenetic affinity [8,9]. Conversely, a novel functional class of 'Trx GPx-like peroxidase (TGPx)' has been proposed to clarify the unique GPx group sharing a common evolutionary origin with the GSH-dependent GPxs [5]. The molecular basis for the differential preference has also been investigated and appeared to involve a 'resolving Cys' within the α2 helix of the Trx-dependent GPxs [5,18,19].

With the accumulation of genomic databases, it has been possible to analyze homologous genes from diverse taxonomical groups. In this context, the evolutionary relationships among the eight GPx families including the complex PHGPx-like proteins were comprehensively examined [5,20]. The proteins isolated from all metazoan species were clearly separated from those of fungi/algae/prokaryotes and plants, and some of algal proteins were dispersed in a distinct group together with the Kinetoplastida GPxs [20]. These analyses demonstrate that PHGPx-like proteins are the most abundant type found in almost all aerobic organisms and considered as an ancestral form of the GPx superfamily [20]. The common ancestor appears to have diverged into GPx7/8 and GPx1/2/3/5/6 groups after being duplicated in the vertebrate lineage [5,20]. The highly polytomic relationships suggest parallel evolutionary pathways for the GPx families following duplication events in the early stage of GPx evolution. Lateral gene transfer has also been introduced as a relevant mechanism for the presence of GPx3-like genes in parasitic nematodes [5]. However, the evolutionary aspects of GPx genes isolated from invertebrates have not yet been fully investigated. The evolutionary relationship between GSH- and Trx-dependent GPxs also remains largely unclear.

Recent molecular phylogeny has positioned the Platyhelminthes into Lophotrochozoa, which forms a monophyletic clade Protostomia with the Ecdysozoa [21,22]. The phylum is composed of markedly diverse species, most of which have established a parasitic life mode within specific host organisms. The tissue-invasive parasites are continuously exposed to oxidative stresses generated by both endogenous and exogenous ROS, which are generated by host immune cells [23,24]. Therefore, parasitic nematodes and platyhelminths might provide good models for the investigation of biochemical and physiological diversification of antioxidant enzymes. Together with the Trx system, GSH-dependent proteins perform central roles in the thiol-disulfide redox homeostasis in these parasites by providing electrons to essential enzymes and by protecting against oxidative stress [25,26]. GPxs homologous to the mammalian plasma GPx (GPx3) and PHGPx have been characterized only from a few filarial nematodes and trematodes, respectively [25,27,28].

The trematode GPxs seem to play specialized roles in their unique biotic environments, in association with sexual reproduction [28,29]. In the present study, we isolated GPx genes in lower animals, especially in platyhelminths and nematodes, and investigated their evolutionary aspects. Almost all of the platyhelminth genes examined displayed a strikingly close relationship to the mammalian PHGPx genes. In addition to the previously described GPx3-like genes, vertebrate-type PHGPx genes were also identified in nematodes. Comparative analyses further indicated that the GPx7 family, which has been known to be specific to vertebrates, already diverged in the Lophotrochozoa, although the gene lineage seems to have been deleted in most of the lower animal genomes. At structural and functional levels, the GSH-dependent platyhelminth and nematode PHGPx-like genes appear to share an evolutionary progenitor with the deuterostomian homologs. The Trx-dependent GPx genes found in the other ecdysozoans may have modularly evolved from another paralogous gene duplicated in a common metazoan/plant ancestor.

## Results

### Isolation of platyhelminth genes encoding GPx

**Figure 1:**
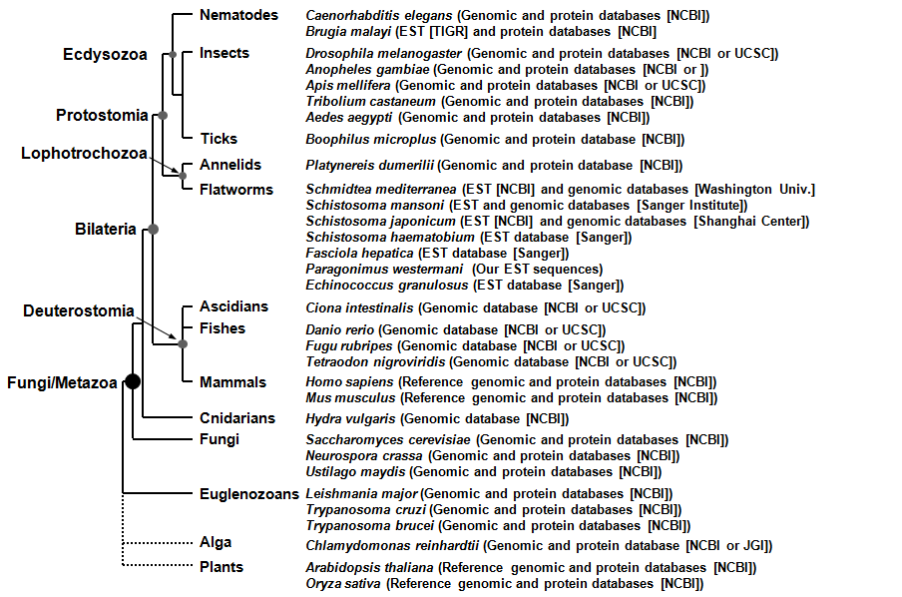
**Organisms used in this study and their phylogenetic relationships**. The rectangular cladogram was constructed based on a recent animal phylogeny [21]. The databases targeted for the screening of GPx homologs are presented in parentheses together with their respective sources, following the scientific names of donor organisms.

The GPx genes were isolated from various platyhelminths via *in silico *screening of their respective genomic/expressed sequence tag (EST) databases (Figure 1). Our preliminary analysis of ESTs obtained from *Paragonimus westermani *adults detected two genes encoding GPx-like proteins (PwGPx1 [GenBank: ABE68811] and PwGPx2 [GenBank: ABE68812]). The *Paragonimus *proteins revealed significant identities to GPx proteins including those of *Schistosoma mansoni *[GenBank: AAU34080, AAC14468]. BLAST searches with these GPx sequences retrieved two homologs from the *S. japonicum *EST database (97,525 ESTs in the GenBank [CV689936, AY223160]), and one from each of the *S. haematobium *(15,000 ESTs; Shaem32f07.q1k, *ShGPx1*) and *Fasciola hepatica *databases (15,000 ESTs; Fhep45e11.q1k, *FhGPx1*) at the Sanger Institute. Screening of the genomic databases for *S. mansoni *(assembly ver. 3.0) and *S. japonicum *(ver. 2.0) revealed that schistosomes possess only two GPx genes corresponded to each of the mRNA sequences (see also [27]). The EST (73,587 entries) and genomic databases (ver. 3.1) of *Schmidtea mediterranea *(Class Turbellaria) contained three paralogous GPx genes (DN292195/DN303738 for *SmedGPx1*, DN310389 for *SmedGPx2*, and DN312778 for *SmedGPx3*). A GPx gene (EGPSgr-5h09.q1k, *EgGPx1*) was also found in the EST database of a parasitic cestode *Echinococcus granulosus *(15,000 ESTs). Searches using human [GenBank: CAA68491, AAP50261] and nematode [GenBank: CAA48882] GPx1/3 lineages showed similar results, although the *E*-values were moderately increased from <3 × 10^-23 ^to <6 × 10^-16^. The BLAST results were supported by the subsequent analyses for the detection of functional domain(s) conserved in these proteins, based on Hidden Markov Models (InterPro IPR000889 family).

### Nucleotide and amino acid sequences of platyhelminth GPx genes

**Figure 2:**
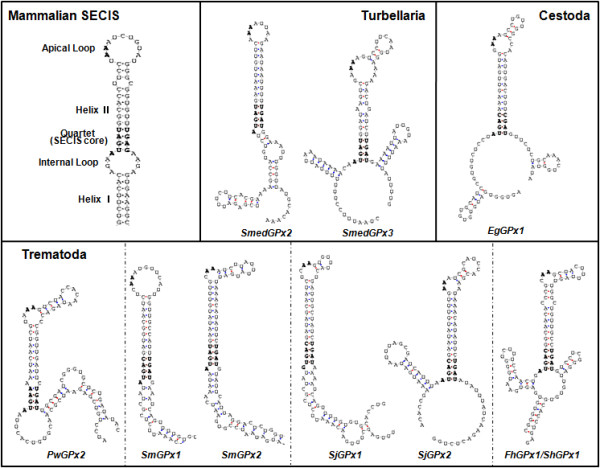
**Selenocysteine insertion sequence (SECIS) motifs found in the platyhelminth GPx genes**. The structures are predicted from the nucleotide sequences corresponding to the 3'-untranslated regions of the respective mRNAs. The typical secondary structure of the mammalian SECIS is also presented for comparison [31].

The majority of the platyhelminth genes isolated in this study encoded a Sec-dependent GPx, as predicted by the detection of a second in-frame TGA codon and a concurrent Sec insertion sequence (SECIS) motif within their 3'-untranslated region (UTR) [27,30]. The motifs conserved the unique secondary structure comprising two helices separated by an internal loop, a SECIS core structure, a quartet located at the base of the second helix, and an apical loop; these were equivalent to those of the mammalian homologs (Figure 2) [31]. The quartets of non-Watson-Crick base pairs were class I (ATGA_AA_GA) in these platyhelminth genes, as were the cases in the mammalian selenoprotein genes. Exceptionally, *SjGPx1 *contained a quartet pattern of class II (GTGA_AA_GA). In contrast, *PwGPx1 *contained a standard codon for Cys (TGC) rather than the Sec codon and the SECIS was not detected within its nucleotide sequence.

The polypeptides encoded by the platyhelminth GPx genes showed a broad range of sequence identity either to one another or to the other eukaryotic PHGPx-like proteins (21–87%). The N-terminal amino acid (aa) extension was hydrophobic in a major fraction of the trematode and planarian proteins (underlined sequences in Additional file 1), while such sequences were not detected in SmGPx1, SjGPx1, and ShGPx1. The presence/absence of the leader sequences would be responsible for the expression of differentially targeted proteins, as was evidenced in the mitochondrial/non-mitochondrial variants of mammalian PHGPxs [32]. The active Cys, Gln, and Trp residues, which participate in the formation of the catalytic-site geometry, were well conserved at their corresponding positions, although the thiol-containing Cys was exclusively replaced by Sec in the platyhelminth and mammalian GPxs (T-1, -2, and -3 in Additional file 1) [19,33]. The Gln and Trp residues were replaced by Met/Leu and Tyr in the *Paragonimus *proteins. The other functional aa involved in the stabilization of the active site, were also detected in their primary structures (C-1 [Asn], -2 [Cys], and -3 [Asn]). The Cys residue, which is pivotal in the interaction of Trx-dependent GPx with Trx, could not be defined in the platyhelminth proteins, as well as in the nematode and mammalian PHGPxs [5,18]. These lower animal GPxs lacked the subunit-interacting domain and PGGG motif found in the tetramer-forming GPxs.

### Expression profiles and preferential electron donor

In trematode parasites, the expression of various GPx genes increased in proportion to the development of donor organisms and showed a specific locality within reproduction-related cells such as vitellocytes [27-29]. Exogenous oxidative chemicals including paraquat, juglone, and H_2_O_2_also induced the trematode genes in a dose-dependent manner [27,28]. *PwGPx *genes exhibited expression patterns identical to those of the *Schistosoma *and *Clonorchis *orthologs, as was evident in RT-PCR and immunohistochemical analyses (unpublished data). The enzymatic activity was not detected in the metacestode stage of *E. granulosus *[34], even though an mRNA sequence (*EgGPx1*) was isolated from the larval stage. We could not currently trace any empirical data demonstrating expression profiles of the antioxidant enzymes in Turbellaria. Given the sexual reproduction mechanism conserved in these platyhelminths [35-37], the planarian genes might have an expression pattern similar to that of the trematode genes.

**Figure 3:**
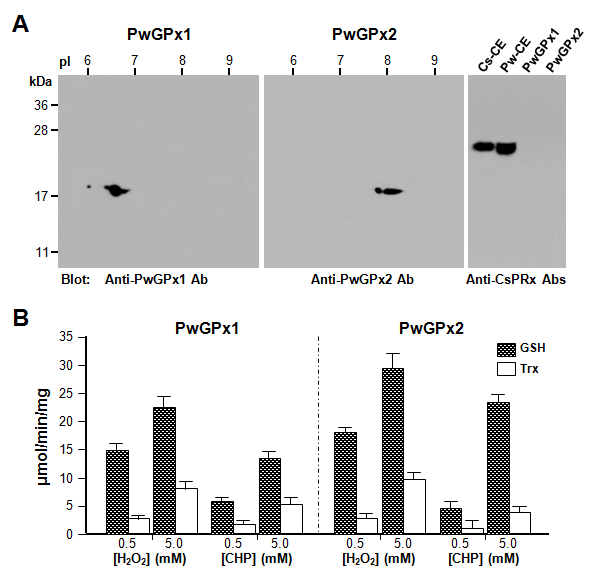
**Biochemical properties of the native PwGPx1 and PwGPx2 proteins**. (A) The partially purified PwGPxs were resolved by 2-dimensional SDS-PAGE (15%, pH 3-10) and transferred onto nylon membranes. The membranes were reacted with the PwGPx-specific mouse antisera (1:2,000 dilutions). One-dimensional blots with PwGPxs (10 μg) and whole protein extracts (40 μg) of *Paragonimus westermani *(Pw-CE) and *Clonorchis sinensis *(Cs-CE) were also examined with a pooled mouse antiserum against *C. sinensis *PRx1 and PRx2 (1: 1,000 dilutions; our unpublished data). (B) The specificity and catalytic efficiency of the proteins purified by a series of gel chromatographies were examined toward catalytic substrates (H_2_O_2 _and cumene hydroperoxide [CHP]) and electron donors (glutathione [GSH] and thioredoxin [Trx]), respectively. The concentrations of GSH and Trx added in the reactions were empirically determined to ensure that the reductants are saturated. The absorbance values of experimental groups were substracted with those of reactions without PwGPxs. The reactions were performed in triplicate and the specific activities in μmol/min/mg were indicated as mean ± standard deviation.

The substrate specificity of schistosome GPxs was likely to be similar to those of mammalian PHGPxs [38], and the Cys motif found in the Trx-dependent GPxs was not detected in the primary structures of platyhelminth proteins analyzed in this study (Additional file 1). The preferential affinity toward electron donors has not been determined empirically with any of the platyhelminth GPx proteins including the well characterized *Schistosoma *and *Clonorchis *enzymes. We examined the catalytic efficiency with the native GPxs partially purified from adult *P. westermani *(Figure 3). The enzymes effectively reduced H_2_O_2 _and cumene hydroperoxide using GSH as an electron donor. The proteins also showed considerable reactivity with Trx, while these activities were lower than those with GSH (15.4–39.4%). The specific activity of GPx proteins decreases dramatically in the range of several tens to several hundred, when provided by an alternative reductant, rather than their preferential electron donor (see [18] and references therein). Therefore, it is possible that the purified *Paragonimus *proteins might have been contaminated with PRxs, although we examined the samples with the *Clonorchis sinensis *PRx-specific antibodies, which were highly cross-reactive against *P. westermani *PRxs (Figure 3A). Regardless of this possibility, however, these observations collectively suggest that the trematode GPxs exhibit a catalytic pattern identical to that of the mammalian PHGPx proteins.

### Phylogenetic analysis of GPx gene families

In addition to the platyhelminth proteins, we retrieved several hundred GPx-like proteins either from the non-redundant GenBank or organism-specific databases (sequence identity: 35–53%, *E*-values < 10^-16^). Proteins with primary structures similar to those of mammalian PHGPxs were found in all major taxa including plants, bacteria, protozoans, fungi, algae, invertebrates, and vertebrates. GPx3-like proteins, which had been likely to be vertebrate-specific, were further found in nematode and tunicate species. The nematode GPx3-like proteins are proposed to have originated from vertebrate hosts by lateral gene transfer [20]. However, the presence of GPx3-like proteins in a tunicate, which has not been described previously, implies that the hypothesis of horizontal gene transfer from vertebrate(s) to nematode(s) needs to be re-examined.

The sequences of 105 proteins were selected for cladistic analyses (Additional file 2). Intra-group divergences inferred from the Jones-Taylor-Thornton (JTT) model were not significantly different between the PHGPx- and GPx1/3-like groups (*P *= 0.053; Mann-Whitney-Wilcoxon test), although the values were highly variable: 0.33–2.37 in the PHGPx-like groups versus 0.31–0.55 in the GPx1/3 groups (bold-face values in Table 1). At the inter-group level, the rates of divergence between a pair of PHGPx and GPx1/3 group were considerably higher than those between a pair of the same GPx family (Table 1). These results suggest that each of the GPx families has diverged during similar evolutionary times and that the GPx3-like proteins are endogenous in nematodes, rather than having originated by lateral gene transfer. To verify these views, we performed in-depth phylogenetic analyses.

**Table 1:** Pairwise matrix of divergence rates among GPx groups based on the JTT model^a^

Group^b^	1	2	3	4	5	6	7	8	9	10	11
1. Trematode GPx	**0.80 ± 0.10**	1.73 ± 0.17	1.61 ± 0.18	1.41 ± 0.17	1.29 ± 0.16	1.30 ± 0.15	1.81 ± 0.25	1.75 ± 0.22	2.11 ± 0.35	2.22 ± 0.35	2.80 ± 0.50
2. Turbellaria GPx		**2.37 ± 0.32**	2.06 ± 0.24	2.08 ± 0.24	1.78 ± 0.19	1.74 ± 0.17	2.20 ± 0.29	2.07 ± 0.22	2.52 ± 0.37	2.83 ± 0.38	3.60 ± 0.54
3. Vertebrate GPx4			**1.50 ± 0.20**	1.78 ± 0.21	1.74 ± 0.23	1.63 ± 0.18	2.08 ± 0.27	1.88 ± 0.22	2.55 ± 0.41	2.53 ± 0.37	3.32 ± 0.53
4. Insect GPx				**1.19 ± 0.13**	1.37 ± 0.17	1.29 ± 0.15	1.93 ± 0.28	1.75 ± 0.21	2.21 ± 0.30	2.44 ± 0.37	2.68 ± 0.37
5. Nematode GPx4					**0.59 ± 0.09**	1.00 ± 0.12	1.65 ± 0.24	1.56 ± 0.20	2.21 ± 0.36	2.23 ± 0.39	2.58 ± 0.44
6. Plant GPx						**0.56 ± 0.06**	1.28 ± 0.18	1.36 ± 0.16	2.06 ± 0.32	2.32 ± 0.37	2.63 ± 0.43
7. Protozoa GPx							**0.33 ± 0.05**	1.82 ± 0.23	2.68 ± 0.49	2.64 ± 0.43	2.98 ± 0.55
8. Fungus GPx								**1.00 ± 0.14**	2.26 ± 0.36	2.34 ± 0.37	2.97 ± 0.50
9. Vertebrate GPx1									**0.47 ± 0.07**	1.37 ± 0.18	2.15 ± 0.33
10. Vertebrate GPx 3										**0.31 ± 0.04**	2.04 ± 0.29
11. Nematode GPx3											**0.55 ± 0.08**

^a^Distance values are presented as mean ± standard error computed by bootstrapping of 1,000 replicates. Bold-face and regular letters indicate intra-group and inter-group rates, respectively.

^b^The proteins were categorized into groups by considering primary structures and taxonomical positions of donor organisms.

**Figure 4:**
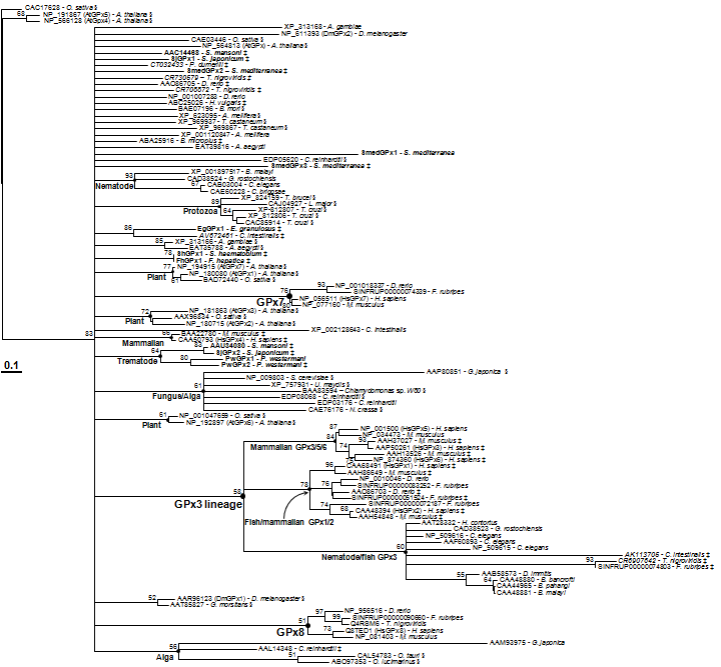
**Maximum likelihood tree of the GPx homologs**. The analysis was conducted on the alignment of amino acid sequences with TREE_PUZZLE. Gaps in the alignment were removed in a pair-wise manner as missing information. The plant PHGPxs [GenBank: CAC17628, NP_191867, NP_566128], which showed the long branches, were taken as the outgroup. The identity of each sequence was provided by a protein identification number followed by the name of donor specie. The platyhelminth genes are presented in the boldface letters and those retrieved from EST databases are marked with the italicized accession numbers of mRNA sequences. The entries with a codon for selenocysteine (Sec) and concurrent Sec insertion sequence within the corresponding mRNA sequences are indicated by ‡. The symbol § marks the proteins with the Cys motif. Quartet support values are presented at each of the major branching points.

The PHGPx-like proteins exhibited a highly polytomic clustering pattern in a maximum likelihood tree constructed by the quart puzzling method of TREE_PUZZLE (Figure 4, the NEWICK-format tree is attached as Additional file 3). The phyletic GPx3-lineage members comprised another polytomic clade. A portion of the orthologous PHGPxs were clustered into well-separated focal clades, in accordance with the taxonomical positions of their donor organisms. The GPx7 and GPx8 proteins, which have been recently detected in vertebrate species, positioned into each of the distinct clades. In protostomians examined, only nematode species harbored the GPx3-like proteins. The neighbor-joining and maximum parsimony trees showed topologies similar to that of the maximum likelihood tree (Additional file 4). The group-specific clustering of PHGPxs was much more substantial in the neighbor-joining tree, albeit the statistical significance of some branching nodes could not be supported by bootstrap analyses. The neighbor-joining tree demonstrated that the GPx7 and GPx8 proteins have diverged from each other after being duplicated in vertebrates. More interestingly, two proteins in *S. mediterranea *(SmedGPx1) and *C. intestinalis *[GenBank: XP_002128643] showed a closer relationship with the vertebrate GPx7/8 lineages. Since the planarian is believed to have maintained its free-living life mode, the lineage members might also have endogenously evolved in the protostomian stage. The members seem to have been deleted in the other protostomian species selected in this study.

The Trx-dependent GPxs with the resolving Cys were largely scattered in plants, algae, protozoans, and insects (marked with § in Figure 4 and Additional file 4), while the Cys residue was substituted by another aa in some of the alga and insect proteins. Conversely, the Sec codon and the associated SECIS motif were recognized as being largely confined to the mRNA sequences of the GSH-dependent lophotrochozoan, ascidian, and vertebrate members (indicated by ‡ in Figure 4). Among the ecdysozoans examined, only an arachnidal tick, *Boophilus microplus*, was found to express the Sec-dependent PHGPx protein [GenBank: ABA25916]. The *C. reinhardtii *[GenBank: AAL14348] and *Hydra vulgaris *[GenBank: ABC25026] proteins also contained Sec in their primary structures. The GPx7/8-like proteins found in *S. mediterranea *and *C. intestinalis *were selenium-independent, consistent with vertebrate proteins [5]. However, the evolutionary events resulting in these biased distributions of proteins with different biochemical properties could not be clearly addressed in the phylogenetic analysis.

### Exon-intron structures of PHGPx homologs

**Figure 5:**
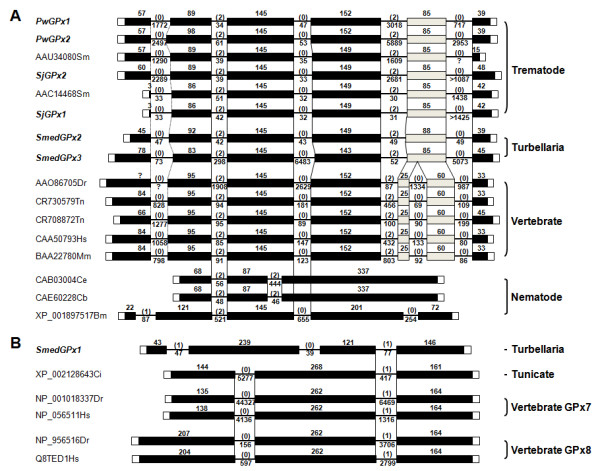
**Exon-intron structures of PHGPx genes putatively encoding glutathione-dependent proteins**. (A) The chromosomal structures of PHGPx genes were constructed by comparing mRNA sequences with their respective chromosomal sequences. ORF regions are presented with solid squares in proportion to their relative sizes and the untranslated regions are shown by open squares with a voluntary length. Intervening introns are indicated by solid lines, with a fixed length. The lengths (bp) of each exon and intron are presented at the corresponding positions. The phase of each intron is shown in parenthesis. Orthologous introns having positions and phases shared among PHGPx genes are indicated by vertical solid lines, while those with a certain degree of ambiguity due to highly divergent aa sequences are marked with dotted lines. The exons of platyhelminth genes, which have been further split into the vertebrate orthologs are indicated by gray-toned squares. (B) The structural conservation patterns of GPx7-and GPx8-like genes are similarly analyzed.

**Figure 6:**
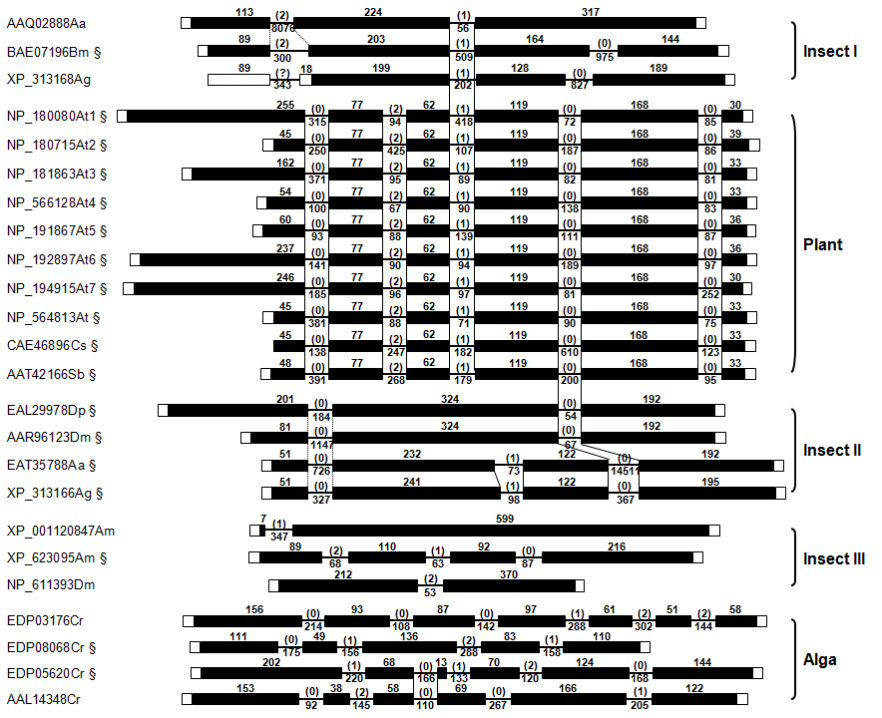
**Genomic organizations of thioredoxin (Trx)-dependent PHGPx genes**. The chromosomal structures of insect and plant genes that encode the Trx-dependent proteins were compared to each other to detect orthologous introns. The insect genes are categorized into three groups according to their conservation patterns. The symbol § marks the gene with the Cys motif in its encoded protein. More detail is presented in the legend to Figure 5.

The polytomic phylogeny of PHGPxs suggested that these proteins have evolved in a modular fashion from multiple genes, while informative diagnostic substitutions could not be detected between the dichotomized GSH- and Trx-dependent proteins, except for the resolving Cys in the major Trx-dependent GPxs (Additional file 2). We examined the presence of orthologous introns in the PHGPx homologs to trace their evolutionary pathways in details. The genomic organizations were predicted by comparing each of the mRNA sequences with its corresponding chromosomal sequence isolated either from DNA databases (Figure 1) or by PCR amplification (*PwGPx*s [GenBank: DQ454159, DQ454160]). The numbers of intron were highly variable in the diverse PHGPx genes, with a gross tendency to increase along with the taxonomical positions of donor organisms (Figures 5 and 6). The genes from free-living nematodes, vertebrates, and plants were split into three, seven, and six exons, respectively. Meanwhile, insect genes displayed highly complicated exon-intron structures with different intron numbers from one to three. Interestingly, the platyhelminth genes displayed an organization pattern comparable to that of mammalian orthologs. A gene isolated from a parasitic nematode *Brugia malayi *was more complex than those of free-living nematodes.

The preservative organization patterns were analyzed by comparing the position of each intron in relation to the aa alignment and their phases. The phases and positions of the respective introns were also conserved in the taxon-specific gene groups. Among the groups, however, orthologous introns shared in the evolutionary terms could be detected on the basis of the biochemical properties of their encoded proteins. The introns were tightly conserved between the Sec- and GSH-dependent platyhelminth and vertebrate genes, except for one intron that was specifically integrated into the vertebrate orthologs. The first intron of *Caenorhabditis *genes was matched to the second one identified in these common decedents. Two introns of the *Brugia *gene were found to be orthologous to the platyhelminth and vertebrate introns (Figure 5A). The GPx7/8-like genes of *S. mediterranea *and *C. intestinalis*, as proposed in the phylogenetic analysis, displayed a different intron conservation pattern (Figure 5B). The exon-intron architecture of the tunicate gene was identical to those of vertebrate GPx7 and GPx8 genes, and the turbellaria gene shared the last intron with them. The complex insect genes, which encode proteins exhibiting strong affinity to Trx, shared an intron with plant GPxs in a dichotomized pattern (Insect I and II in Figure 6). In addition to these clonal genes, GPx genes with unique exon-intron structures were also retrieved from the insect genomes (grouped into Insect III in Figure 6). The *C. reinhardtii *genome possessed genes with each of the unique number and position of intervening intron, including the gene encoding a Sec-dependent protein [GenBank: AAL14348]. The architectural conservation patterns showed weak relations to the presence of resolving Cys in the insect and alga genes (genes containing the motif were marked with § in Figure 6). The phyletic GPx1 and GPx3 members had their respective intron-sharing patterns in the genomic structures, while those were clearly distinguished from the chromosomal organizations of PHGPx members (Additional file 5).

## Discussion

In this study, we described a comparative analysis of GPx genes including platyhelminth orthologs such as the human lung fluke and freshwater planarian genes, for the purpose of predicting their evolutionary pathways. The GPx homologs displayed diversity in the copy number among the eukaryotic species, which suggests that independent multiplication events have occurred in each of the taxa. In nematodes and deuterostomians, a couple of duplicated genes have diverged further into GPx1 and GPx3 lineages, respectively, while all GPx paralogs have preserved the structural properties similar to that of the mammalian PHGPx genes in the other invertebrate clades. A GPx7/8-like gene previously shown to be vertebrate-specific was isolated from the genomes of a tunicate and turbellaria. The spatiotemporal expressions of GPx genes were found to be specific to each of the duplicated copies in plants and vertebrates, which demonstrate functional diversification among them [4,9]. However, the trematode gene inductions were concentrated in the reproduction-related vitellocytes contained within vitelline follicles and eggs. This fact might suggest that the duplication events have been forced to increase genetic redundancy and/or protein levels, rather than to increase the size of isoenzyme pools with distinct tissue expression profiles, at least in the trematode species.

The substrate specificity of GPx-like proteins has been debatable not only because the parasitic nematode proteins are not enzymatically active toward H_2_O_2 _but also because PHGPxs show a variation against diverse catalytic substrates. The trematode PHGPxs reduced H_2_O_2 _much more effectively than cumene hydroperoxide, as was determined with the *Paragonimus *(Figure 3) and *Schistosoma *proteins [38], whereas plant homologs preferred the organic peroxide [9]. Interestingly, the GSH-dependent incapability of plant PHGPxs in reduction of H_2_O_2 _was reversed when Trx was provided as an alternative electron donor [10]. These results suggest that the GPx family genes have functionally diverged according to the biochemistry of their action environments. The active site geometry is modified in response to the binding of GSH or Trx [33]. The vitellocyte-specific platyhelminth genes were inducible by exogenous stimuli with the soluble hydroperoxide and redox-cycling drugs, which can cross cell boundaries and trigger the conversion of oxygen into O_2_^- ^[39]. The increased GPx activity against these chemicals seems to result indirectly from the accumulation of H_2_O_2 _and/or lipid peroxidation within the target cells/organs, due to O_2_^- ^overproduction. Alternatively, their responsiveness upon these stimuli could be a simple consequence of the coordinated regulation of phase I oxidative enzymes via the Nrf2-mediated signaling pathway [40], given the fact that the specific role of trematode GPx proteins is highly associated with sexual reproduction [28,29].

The majority of the mammalian GPxs contain Sec at their catalytic site, which is co-translationally inserted in response to a UGA codon, the stop signal in the standard genetic code. The alternative decoding of the opal codon depends on a *cis*-factor (SECIS) located in the 3'-UTR of selenoprotein genes [30]. In our analysis, the distribution of selenium-dependent GPxs (sGPxs) was biased toward platyhelminth and deuterostomian species, with a few exceptions: proteins isolated from an annelid [GenBank: ABA25916], alga [GenBank: AAL14348], and cnidarian [GenBank: ABC25026]. Of the platyhelminth enzymes obtainable, PwGPx1 and SmedGPx1 (GPx7-like protein) were determined to be selenium-independent GPx (siGPx). The Sec codon was replaced by a typical Cys codon in the nematode homologs, although nematode species also contained selenoproteins such as Trx reductase and/or SelK [41]. It has been suggested that siGPx proteins have emerged independently from sGPx counterparts during evolution, in response to the increased atmospheric oxygen concentration [16,42]. Conversely, a recent comparative analysis predicted the progressive acquisition of Sec by Cys-based proteins in a series of donor organisms [5]. The siGPx in protozoans, fungi, and plants, and the biased distribution of sGPx within a narrow range of taxonomical clades seems to support the Cys-based evolutionary route of GPx proteins. The highly conserved genomic architecture among Sec-dependent genes can be taken as evidence for the suggestion (Figure 5). siGPx has been postulated either to comprise a second-line defense or to cooperate with sGPx in as yet-unknown and novel manner, to cope with cellular oxidative stresses [43]. In plants and lower animals, PRx constitutes a major antioxidant system against ROS-derived damage and the platyhelminth GPx seems to be involved in a specialized physiological function(s) [9,10,27,28]. Therefore, it is possible that siGPx carries out its role in a specific microenvironment with an optimal pH for the thiol group, along with the diversification of its donor organism. The siGPxs of filarial nematodes, of which functions are mainly confined to the cuticular matrix, may provide another example of this suggestion [44]. The induction level of PwGPx1 was higher than that of PwGPx2 during the development of *P. westermani *and against exogenous oxidative stresses, while their histological distributions were almost identical (data not shown). Currently, it is not clear whether this phenomenon is related to their differential reactivity or it is an indication of functional and/or histological diversification within each of their respective subcellular micro-niches.

Together with the selenium dependency, the categories of GPx isoenzymes also revealed taxonomical biases along with their donor organisms; the GPx3-like proteins were detected exclusively in the deuterostomian and nematode species, whereas those with a phylogenetic linkage to PHGPx were ubiquitously isolated across taxa from plants to vertebrates. Since the complete or draft whole genome sequences with a considerable coverage (>7) have been comprehensively screened via a series of BLAST searches (Figure 1; see also [9,15,27]), the absence of genes encoding GPx3-like proteins appears apparent in the plant and invertebrate genomes selected in this study. The free-living and pytoparasitic nematodes [45], of which whole genome or comprehensive EST databases are available, contained both of the GPx lineages. The GPx3-like filarial proteins were identified empirically by analyzing the major cuticular glycoproteins [46]. Recently, we detected a PHGPx-like gene from the *B. malayi *DNA database [GenBank: XP_001897517]. Genes highly homologous to the vertebrate GPx7/8 genes were also detected exclusively in the free-living turbellaria and tunicate (Figures 4 and 5B). Therefore, it is evident that there have been a series of selection pressures to drive the mosaic distributions of the GPx3- and GPx7-lineage genes across taxa, which was similarly observed in the DNA methyltranferase gene homologs [47], although the mechanism(s) in the selection of genes for deletion/maintenance waits for further investigation.

The tetrameric GPx1/3-lineage proteins were phylogenetically related to one another in the cladistic analysis and their chromosomal genes showed clonal conservation patterns (Figure 4 and Additional file 4). On the contrary, the phylogeny and genomic structures of PHGPx genes were found to be multifaceted. The aa sequences of PHGPxs were well aligned throughout the whole polypeptides, except for the extreme N-terminal segments, and gaps resulted from indels of codon were not prominent (Additional file 2). The polymorphic N-terminal regions were intimately related to the evolutionary acquisition/loss of regulatory signal within certain members (Additional file 1). Despite the tight sequence conservation, the hypervariable genomic structures of these genes exhibited a discontinuous conservation patterns according to the taxonomical distributions and/or biochemical properties of protein products (Figures 5 and 6). Whether we accept the "intron-early" or the "intron-late" hypothesis concerning the origin of spliceosomal introns, it is clear that an orthologous intron can be taken as a footprint revealing a common ancestor during evolution of the corresponding genes [48]. Therefore, each of the genes encoding GSH- and Trx-dependent proteins might have evolved along separate pathways from a certain evolutionary time, although the enzymatic properties of the nematode PHGPxs have not yet been examined empirically. Compared to the tightly conserved introns among the platyhelminth and vertebrate orthologs, intron preservation was much more complicated in the Trx-dependent insect and plant genes. The third intron of plant genes was orthologous to the second intron of some insect genes (Group I) and the forth intron was shared with the other insect genes (Group II), while a series of insect genes (Group III) with uniquely integrated introns were further recognized. The aa sequences of plant exons 3, 4, and 5 displayed identity values of 47.8, 59.8, and 53.3%, respectively, to the corresponding regions of Group I insect genes, and 49.8, 41.2, and 64.0%, respectively, to those of Group II insect genes. These collective results suggest a probable recombination event at a region matched to the end of current exon 4, which had occurred between two paralogs in a primordial genome that evolved into plants. After gaining additional introns, the gene might have undergone genic/chromosomal multiplications. Alternatively, each of the diverse insect genes might have unique exon-intron remodeling process mediated by a gene's recombination with a cDNA generated by reverse transcription of the respective mRNA [49]. Further information on the intermediate metazoan genes would be informative to address this intriguing issue.

The fully diverged mammalian genes were present as a single genomic copy, whereas the PHGPx- or GPx3-like genes had multiple copies in the respective genomes of the other metazoans and plants. Together with the polytomic relationships and differentiated genomic organizations, this fact is likely to provide additional evidence for the modular evolutionary pathways of GPx gene family from multiple intermediates. The presence of GPx3 in the soil-borne and pytoparasitic nematodes, and GPx7 in the free-living planarian and tunicate, respectively, would reduce the possibility of lateral gene transfer between taxa. Each of the primordial paralogs duplicated from a common ancestor seems to have been subject to independent exon-intron remodeling processes such as homologous recombination and integration of intronic sequences, along with their taxonomical lineages. A series of the intermediate genes might have diverged either to GPx3-like genes by gaining exonic nucleotides corresponding to the subunit interaction domain or to GPx7 homologs, while they underwent lineage-specific expansion or deletion in the metazoan animals. The GPx1/2 genes are likely to have evolved from the retrointegration of GPx3-like intermediate aided by retrotransposon-encoded proteins, which was followed by acquisition of introns, during an early evolutionary period of the Vertebrata. The evolutionary pathways for the GSH- and Trx-dependent GPxs appear be separated from the common eukaryotic ancestor. The Trx-dependent GPxs without the resolving Cys in insects and algae would be acquired by the relaxation of selective constraints and/or by the adaptation to the micro-environments in their specific action sites. Further studies on biochemical properties with the individual insect GPxs and detection of a second Cys motif, if any, would be helpful to gain more insight into the complex evolution of PHGPx-like proteins.

## Conclusion

Our study on the comparative analysis of GPx gene families suggests that these genes have modularly evolved from multiple intermediates, which accompanied the structural diversification and sporadic expansion/deletion. In addition, the ubiquitous PHGPx genes with different biochemical properties are likely to have been separated in an early evolutionary time. The dichotomized evolutionary pathways were traceable by considering structural conservation patterns in the chromosomal genes. Multiple GPx genes have tissue-specific implications in mammals and plants. However, the duplication events appear to be intimately related to an increase in the genetic repertoire of the antioxidant enzyme, at least in the platyhelminth species. The modular evolution of GPx genes in association with their biochemical properties may provide a molecular basis for understanding the detailed physiological implications of this antioxidant enzyme system. The complexity in genomic structures of platyhelminth genes, which is comparable to that of mammalian genes, further suggests that these organisms comprise an informative model system in elucidating the driving-forces and courses of genomic complexity during eukaryotic evolution.

## Methods

### *In silico *isolation of GPx genes from platyhelminth species

Two novel genes encoding GPx proteins (*PwGPx1 *and *PwGPx2*) were isolated from an EST dataset of *P. westermani *[50]. The *Paragonimus *genes, together with those of *S. mansoni *[GenBank: L37762, AY729668] were used as queries in the homology searches to retrieve their orthologous genes. Genomic and/or EST databases specific to platyhelminths were screened via stand-alone BLAST searches, including *S. mansoni, S. haematobium, F. hepatica, E. granulosus *(Sanger Institute; ), *S. japonicum *(Shanghai Center for Life Science and Biotechnology Information; ), and *S. mediterranea *(Genome Sequencing Center of Washington University; ) (Figure 1). The GenBank DNA/protein databases at the National Center for Biotechnology Information (NCBI; ) were also selected for the screening. GPx proteins isolated from human (GPx1 and GPx3 [GenBank: CAA68491, AAP50261]) and filarial nematode (*Brugia pahangi *[GenBank: CAA48882]) were employed as the other queries during the analyses.

### Sequence analysis

The coding profiles and patterns of sequence similarity of the retrieved mRNA sequences were analyzed using the ORF Finder and BLAST programs equipped at the NCBI. The similarity pattern of each sequence was verified by a subsequent analysis based on the Hidden Markov Models using InterProScan (). The SECIS motif was predicted using the SECISearch program (ver. 2.19; ). The putative hydrophobic signal peptide was analyzed by the SignalP program (). Chromosomal segments homologous to the *PwGPx *cDNAs were amplified from the *P. westermani *genome by long-range PCR using the gene-specific primer pairs, which had been designed from the nucleotide sequences within both ends of the cDNAs (5'-GGTACCAACAGTGACGGTTTGATTTTCTAACACC-3' and 5'-GACAGGCCTGGAGGTGAATTGATGAGAGTGAACC-3' for *PwGPx1*; 5'-GGAACATCGAAGGTGGTTTGAAAAAGGTCAACTTC-3' and 5'-CTTTACTCACAAACTACTGTTGCAATAATAGTAACGTC-3' for *PwGPx2*). PCR was conducted with the LA *Taq *system (Takara, Shiga, Japan) and the resulting PCR products were cloned into the pGEM-T Easy vector (Promega, Madison, WI, USA) for sequencing. The nucleotide sequences were determined from both strands using a BigDye Terminator Cycle Sequencing Core Kit (Perkin Elmer, Foster City, CA, USA) and an automated ABI PRISM 377A DNA Sequencer (Applied Biosystems, Foster City, CA, USA). The mRNA sequences were aligned with their corresponding genomic sequences by considering the general rule for nucleotides tightly conserved in the exon-intron boundary [51], after which their chromosomal structures were determined [GenBank: DQ454159, DQ454160].

### Enzymatic characterization of native PwGPx proteins

The native proteins were purified from an adult worm extract using an AKTA fast-performance liquid chromatography (Superdex 75), followed by DEAE-anion exchange chromatography (Amersham Pharmacia Biotech, Uppsala, Sweden), by monitoring GPx activity. The proteins were also examined by 1-dimensional (1-D) and/or 2-D Western blotting employing mouse antisera specific to the recombinant forms of the PwGPx proteins. The purified GPx proteins were dialyzed against PBS (pH 7.2) overnight at 4°C. The specific activity of the native proteins was examined by the reduction of H_2_O_2 _or cumene hydroperoxide (Sigma-Aldrich, St. Louis, MO, USA) in the presence of GSH and *Escherichia coli *glutathione reductase (GR), as described previously [38,52]. The 200 μl reaction mixtures contained 5 mM potassium phosphate, 1 mM GSH, 0.1 unit of GR, 0.1 mM NADPH, 1 mM EDTA, and 1 μM of PwGPx. The reagents were pre-warmed to room temperature just prior to use in the reaction. After adding the substrate, the NADPH oxidation level was monitored at *A*_340 _for 5 min with a spectrophotometer. A series of mixtures containing 10 μM *E. coli *Trx and 0.1 unit of thioredoxin reductase (TR) instead of GSH and GR were also assayed.

### Phylogenetic Analysis

A total of 105 members that were assigned separately into the eight GPx families, were finally selected during the BLAST searches, by considering both the identity values and taxonomical distributions of donor organisms. The aa sequences were aligned using ClustalX and optimized using GeneDoc. The alignment was used as an input to obtain a maximum likelihood tree using the quartet puzzling algorithm implanted in TREE_PUZZLE (ver. 5.2) [53]. The options were selected to use the JTT model for the aa substitution [54], estimation of aa frequencies from the dataset, gamma distribution model for rate heterogeneity (the parameter alpha was estimated from the input data), eight gamma rate categories, and 50,000 puzzling steps. Indels between pairs of sequences were regarded as missing data. Distances between pairs of protein sequences were calculated according to the JTT model and corrected for the gamma distribution of evolutionary rates. The standard errors were computed by the bootstrapping of 1,000 replicates. The sequence alignment was also applied in phylogenetic analyses using the neighbor-joining (PROTDIST and NEIGHBOR) and maximum parsimony (PROTPARS) programs of the PHYLIP package (ver. 3.6b). The statistical significance of each branching point in the resulting trees was evaluated using 1,000 random samplings of the input alignment by the SEQBOOT program.

## Abbreviations

aa: amino acid; EST: expressed sequence tag; GPx: glutathione peroxidase; GSH: glutathione; PHGPx: phospholipid hydroperoxide glutathione peroxidase; PRx: peroxiredoxin; ROS: reactive oxygen species; Sec: selenocysteine; SECIS: Sec insertion sequence; siGPx: selenium-independent GPx; sGPx: selenium-dependent GPx; UTR: untranslated region.

## Authors' contributions

YAB contributed to the experimental design, sequence analyses, sequence alignments and phylogenetic analyses, and drafted the manuscript. GBC performed experiments regarding isolation of the *P. westermani GPx *genes. SHK examined the biochemical properties of the trematode proteins. YGZ was involved in interpretation of bioinformatics data. YK oversaw the research project, contributed in its design and participated in editing the manuscript. All authors read and approved the final manuscript.

## Supplementary Material

Additional file 1 **Comparison of primary structures between the platyhelminth GPxs and their orthologs**. The deduced amino acid sequences were aligned using the ClustalX program, then optimized using GeneDoc. The identical amino acids in the alignment are highlighted in black, while similar residues are shown in gray. Three well-conserved domains found in the catalytic sites are double underlined. The functional amino acid residues engaged in the formation of the catalytic-site geometry are marked as T-1, T-2 and T-3. The highly conserved amino acids of GPx families are indicated by C-1, C-2, and C-3. The abbreviation 'U' in the functional domain represents selenocysteine. The putative target signals are underlined. The Cys motif found in Trx-dependent GPx is distinguished by a box. Click here for file

Additional file 2 **Amino acid sequences**. Multiple alignment of amino acid sequences of GPx proteins used in the phylogenetic analyses. Click here for file

Additional file 3 **Tree Puzzle**. Tree of GPx proteins in NEWICK format. Click here for file

Additional file 4 **The phylogeny of GPx proteins by neighbor-joining algorithm**. The tree was rooted with the plant GPxs [GenBank: CAC17628, NP_191867, NP_566128]. The identity of each analyzed sequence was distinguished by a protein identification number followed by donor species name. The platyhelminth genes are presented in the boldface letters and those retrieved from EST databases are marked with the italicized accession numbers for the mRNA sequences. The entries with a codon for selenocysteine (Sec) and concurrent Sec insertion sequence within the corresponding mRNA sequence are indicated by ‡. The symbol § marks the proteins with the Cys motif. The bootstrapping values with 1,000 replicates are presented at each of the major branching points. To evaluate the statistical significance of each node, bootstrap support values obtained with maximum parsimony algorithm are also presented. Click here for file

Additional file 5 **Exon-intron structures of the GPx1/3-like genes**. The genomic organization patterns were compared with one another to examine the degree of structural conservation. The open reading frame (ORF) regions are presented with black or gray-toned squares in proportion to their relative sizes and the untranslated regions are shown by open squares with a voluntary length. White boxes in the ORF marke the three well-conserved catalytic sites. The lengths of each exon (in bp) are presented at the top. The intervening introns are removed for simplicity of illustration, while their phases are marked in parentheses at the corresponding positions. The orthology among introns is indicated by vertical dotted lines. Click here for file

## References

[B1] Thannickal VJ, Fanburg BL (2000). Reactive oxygen species in cell signaling. Am J Physiol Lung Cell Mol Physiol.

[B2] Jackson MJ (2005). Reactive oxygen species and redox-regulation of skeletal muscle adaptations to exercise. Philos Trans R Soc Lond B Biol Sci.

[B3] Sies H (1993). Strategies of antioxidant defense. Eur J Biochem.

[B4] Arthur JR (2000). The glutathione peroxidases. Cell Mol Life Sci.

[B5] Toppo S, Vanin S, Bosello V, Tosatto SCE (2008). Evolutionary and structural insights into the multifaceted glutathione peroxidase (Gpx) superfamily. Antioxid Redox Signal.

[B6] Grossmann A, Wendel A (1983). Non-reactivity of the selenoenzyme glutathione peroxidase with enzymatically hydroperoxidized phospholipids. Eur J Biochem.

[B7] Imai H, Nakagawa Y (2003). Biological significance of phospholipid hydroperoxide glutathione peroxidase (PHGPx, GPx4) in mammalian cells. Free Radic Biol Med.

[B8] Rouhier N, Jacquot JP (2005). The plant multigenic family of thiol peroxidases. Free Radic Biol Med.

[B9] Navrot N, Collin V, Gualberto J, Gelhaye E, Hirasawa M, Rey P, Knaff DB, Issakidis E, Jacpuot JP, Rouhier N (2006). Plant glutathione peroxidases are functional peroxiredoxins distributed in several subcellular compartments and regulated during biotic and abiotic stresses. Plant Physiol.

[B10] Herbette S, Lenne C, Leblanc N, Julien JL, Drevet JR, Roeckel-Drevet P (2002). Two GPx-like proteins from *Lycopersicon esculentum *and *Helianthus annuus *are antioxidant enzymes with phospholipid hydroperoxide glutathione peroxidase and thioredoxin peroxidase activities. Eur J Biochem.

[B11] Jung BG, Lee KO, Lee SS, Chi YH, Jang HH, Kang SS, Lee K, Lim D, Yoon SC, Yun DJ, Inoue Y, Cho MJ, Lee SY (2002). A Chinese cabbage cDNA with high sequence identity to phospholipid hydroperoxide glutathione peroxidases encodes a novel isoform of thioredoxin-dependent peroxidase. J Biol Chem.

[B12] Sztajer H, Gamain B, Aumann KD, Slomianny C, Becker K, Brigelius-Flohé R, Flohé L (2001). The putative glutathione peroxidase gene of *Plasmodium falciparum *codes for a thioredoxin peroxidase. J Biol Chem.

[B13] Missirlis F, Rahlfs S, Dimopoulos N, Bauer H, Becker K, Hilliker A, Phillips JP, Jäckle H (2003). A putative glutathione peroxidase of *Drosophila *encodes a thioredoxin peroxidase that provides resistance against oxidative stress but fails to complement a lack of catalase activity. Biol Chem.

[B14] Tanaka T, Izawa S, Inoue Y (2005). GPX2, encoding a phospholipid hydroperoxide glutathione peroxidase homologue, codes for an atypical 2-Cys peroxiredoxin in *Saccharomyces cerevisiae*. J Biol Chem.

[B15] Corona M, Robinson GE (2006). Genes of the antioxidant system of the honey bee: annotation and phylogeny. Insect Mol Biol.

[B16] Dayer R, Fischer BB, Eggen RI, Lemaire SD (2008). The peroxiredoxin and glutathione peroxidase families in *Chlamydomonas reinhardtii*. Genetics.

[B17] Holland D, Ben-Hayyim G, Faltin Z, Camoin L, Strosberg AD, Eshdat Y (1993). Molecular characterization of salt-stress-associated protein in citrus: protein and cDNA sequence homology to mammalian glutathione peroxidases. Plant Mol Biol.

[B18] Maiorino M, Ursini F, Bosello V, Toppo S, Tosatto SCE, Mauri P, Becker K, Roveri A, Bulato C, Benazzi L, De Palma A, Flohé L (2007). The thioredoxin specificity of Drosophila GPx: a paradigm for a peroxiredoxin-like mechanism of many glutathione peroxidases. J Mol Biol.

[B19] Tosatto SCE, Bosello V, Fogolari F, Mauri P, Roveri A, Toppo S, Flohé L, Ursini F, Maiorino M (2008). The catalytic site of glutathione peroxidases. Antioxid Redox Signal.

[B20] Margis R, Dunand C, Teixeira FK, Margis-Pinheiro M (2008). Glutathione peroxidase family – an evolutionary overview. FEBS J.

[B21] Halanych KM (2004). The new view of animal phylogeny. Annu Rev Ecol Evol Syst.

[B22] Philippe H, Lartillot N, Brinkmann H (2005). Multigene analyses of bilaterian animals corroborate the monophyly of Ecdysozoa, Lophotrochozoa and Protostomia. Mol Biol Evol.

[B23] Selkirk ME, Smith VP, Thomas GR, Gounaris K (1998). Resistance of filarial nematode parasites to oxidative stress. Int J Parasitol.

[B24] Zelck UE, Von Janowsky B (2004). Antioxidant enzymes in intramolluscan *Schistosoma mansoni *and ROS-induced changes in expression. Parasitology.

[B25] Henkle-Dührsen K, Kampkötter A (2001). Antioxidant enzyme families in parasitic nematodes. Mol Biochem Parasitol.

[B26] Salinas G, Selkirk ME, Chalar C, Maizels RM, Fernández C (2004). Linked thioredoxin-glutathione systems in platyhelminths. Trends Parasitol.

[B27] Sayed AA, Cook SK, Williams DL (2006). Redox balance mechanisms in *Schistosoma mansoni *rely on peroxiredoxins and albumin and implicate peroxiredoxins as novel drug targets. J Biol Chem.

[B28] Cai GB, Bae YA, Kim SH, Sohn WM, Lee YS, Jiang MS, Kim TS, Kong Y (2008). Vitellocyte-specific expression of phospholipid hydroperoxide glutathione peroxidases in *Clonorchis sinensis*. Int J Parasitol.

[B29] Roche C, Liu JL, LePresle T, Capron A, Pierce RJ (1996). Tissue localization and stage-specific expression of the phospholipid hydroperoxide glutathione peroxidase of *Schistosoma mansoni*. Mol Biochem Parasitol.

[B30] Stadtman TC (1996). Selenocysteine. Annu Rev Biochem.

[B31] Kryukov GV, Castellano S, Novoselov SV, Lobanov AV, Zehtab O, Guigó R, Gladyshev VN (2003). Characterization of mammalian selenoproteomes. Science.

[B32] Arai M, Imai H, Sumi D, Imanaka T, Takano T, Chiba N, Nakagawa Y (1996). Import into mitochondria of phospholipid hydroperoxide glutathione peroxidase requires a leader sequence. Biochem Biophys Res Commun.

[B33] Epp O, Ladenstein R, Wendel A (1983). The refined structure of the selenoenzyme glutathione peroxidase at 0.2-nm resolution. Eur J Biochem.

[B34] Salinas G, Fernández V, Fernández C, Selkirk ME (1998). *Echinococcus granulosus *: cloning of a thioredoxin peroxidase. Exp Parasitol.

[B35] Smyth JD, Halton DW (1983). The physiology of trematodes.

[B36] Smyth JD, McManus DP (1989). The physiology and biochemistry of cestodes.

[B37] Sánchez Alvarado A (2003). The freshwater planarian Schmidtea mediterranea: embryogenesis, stem cells and regeneration. Curr Opin Genet Dev.

[B38] Maiorino M, Roche C, Kiess M, Koenig K, Gawlik D, Matthes M, Naldini E, Pierce R, Flohe L (1996). A selenium-containing phospholipid-hydroperoxide glutathione peroxidase in *Schistosoma mansoni*. Eur J Biochem.

[B39] O'Brien PJ (1991). Molecular mechanisms of quinone cytotoxicity. Chem Biol Interact.

[B40] Rushmore TH, Kong AN (2002). Pharmacogenomics, regulation and signaling pathway of phase I and II drug metabolizing enzymes. Curr Drug Metab.

[B41] Taskov K, Chapple C, Kryukov GV, Castellano S, Lobanov AV, Korotkov KV, Guigó R, Gladyshev VN (2005). Nematode selenoproteome: the use of the selenocysteine insertion system to decode one codon in an animal genome?. Nucleic Acids Res.

[B42] Copeland PR (2005). Making sense of nonsense: the evolution of selenocysteine usage in proteins. Genome Biol.

[B43] Utomo A, Jiang X, Furuta S, Yun J, Levin DS, Wang YC, Desai KV, Green JE, Chen PL, Lee WH (2004). Identification of a novel putative non-selenocysteine containing phospholipid hydroperoxide glutathione peroxidase (NPGPx) essential for alleviating oxidative stress generated from polyunsaturated fatty acids in breast cancer cells. J Biol Chem.

[B44] Cookson E, Blaxter ML, Selkirk ME (1992). Identification of the major soluble cuticular glycoprotein of lymphatic filarial nematode parasites (gp29) as a secretory homolog of glutathione peroxidase. Proc Natl Acad Sci USA.

[B45] Jones JT, Reavy B, Smant G, Prior AE (2004). Glutathione peroxidases of the potato cyst nematode *Globodera rostochiensis*. Gene.

[B46] Cookson E, Tang L, Selkirk ME (1993). Conservation of primary sequence of gp29, the major soluble cuticular glycoprotein, in three species of lymphatic filariae. Mol Biochem Parasitol.

[B47] Ponger L, Li WH (2005). Evolutionary diversification of DNA methyltransferases in eukaryotic genomes. Mol Biol Evol.

[B48] Jeffares DC, Mourier T, Penny D (2006). The biology of intron gain and loss. Trends Genet.

[B49] Logsdon JM, Stoltzfus A, Doolittle WF (1998). Molecular evolution: recent cases of spliceosomal intron gain?. Curr Biol.

[B50] Bae YA, Kim SH, Cai GB, Lee EG, Kim TS, Agatsuma T, Kong Y (2007). Differential expression of *Paragonimus westermani *eggshell proteins during the developmental stages. Int J Parasitol.

[B51] Mount SM (1982). A catalogue of splice junction sequences. Nucleic Acids Res.

[B52] Sekiya M, Mulcahy G, Irwin JA, Stack CM, Donnelly SM, Xu W, Collins P, Dalton JP (2006). Biochemical characterization of the recombinant peroxiredoxin (FhePrx) of the liver fluke, *Fasciola hepatica*. FEBS Lett.

[B53] Schmidt HA, Strimmer K, Vingron M, von Haeseler A (2002). TREE-PUZZLE: maximum likelihood phylogenetic analysis using quartets and parallel computing. Bioinformatics.

[B54] Jones DT, Taylor WR, Thornton JM (1992). The rapid generation of mutation data matrices from protein sequences. Comput Appl Biosci.

