# Supplementary figures and images for: Modular evolution of glutathione peroxidase genes in association with different biochemical properties of their encoded proteins in invertebrate animals
Source: BMC Evol Biol. 2009 Apr 6;9:72. doi: 10.1186/1471-2148-9-72 (PMC2679728; doi:10.1186/1471-2148-9-72)

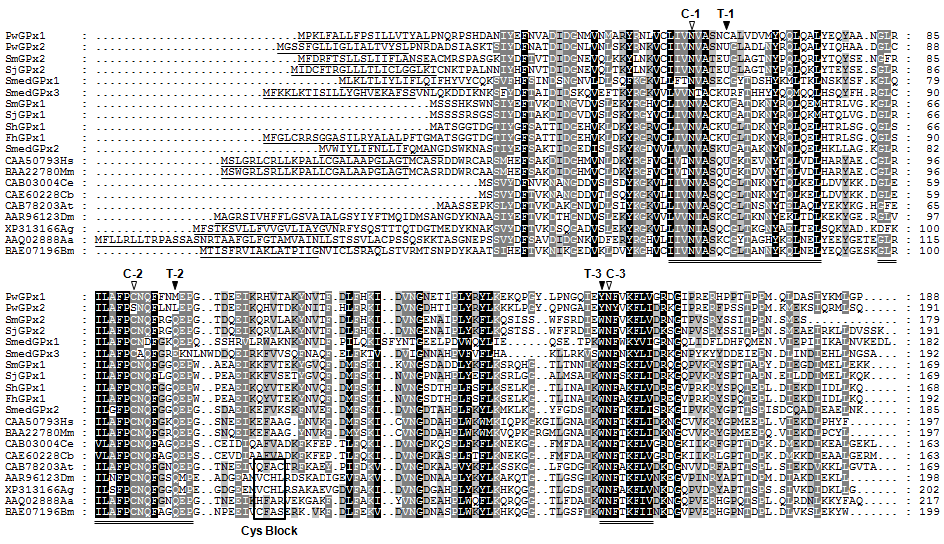

Supplement: Additional file 1 — Comparison of primary structures between the platyhelminth GPxs and their orthologs. The deduced amino acid sequences were aligned using the ClustalX program, then optimized using GeneDoc. The identical amino acids in the alignment are highlighted in black, while similar residues are shown in gray. Three well-conserved domains found in the catalytic sites are double underlined. The functional amino acid residues engaged in the formation of the catalytic-site geometry are marked as T-1, T-2 and T-3. The highly conserved amino acids of GPx families are indicated by C-1, C-2, and C-3. The abbreviation 'U' in the functional domain represents selenocysteine. The putative target signals are underlined. The Cys motif found in Trx-dependent GPx is distinguished by a box. [file 1471-2148-9-72-S1.tiff]

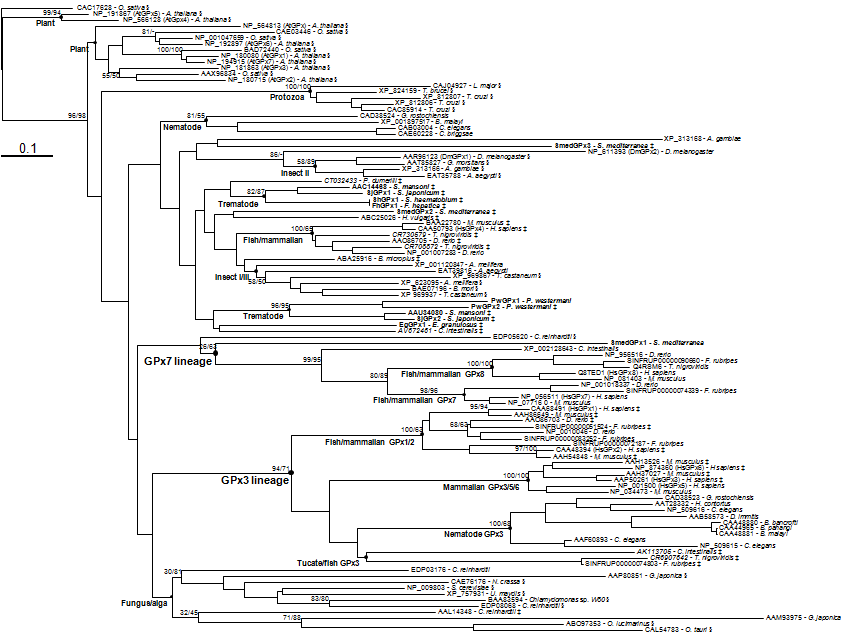

Supplement: Additional file 4 — The phylogeny of GPx proteins by neighbor-joining algorithm. The tree was rooted with the plant GPxs [GenBank: CAC17628, NP_191867, NP_566128]. The identity of each analyzed sequence was distinguished by a protein identification number followed by donor species name. The platyhelminth genes are presented in the boldface letters and those retrieved from EST databases are marked with the italicized accession numbers for the mRNA sequences. The entries with a codon for selenocysteine (Sec) and concurrent Sec insertion sequence within the corresponding mRNA sequence are indicated by ‡. The symbol § marks the proteins with the Cys motif. The bootstrapping values with 1,000 replicates are presented at each of the major branching points. To evaluate the statistical significance of each node, bootstrap support values obtained with maximum parsimony algorithm are also presented. [file 1471-2148-9-72-S4.tiff]

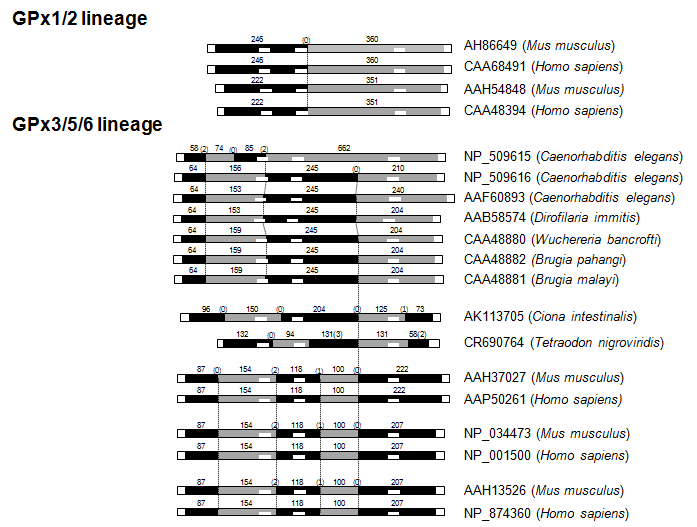

Supplement: Additional file 5 — Exon-intron structures of the GPx1/3-like genes. The genomic organization patterns were compared with one another to examine the degree of structural conservation. The open reading frame (ORF) regions are presented with black or gray-toned squares in proportion to their relative sizes and the untranslated regions are shown by open squares with a voluntary length. White boxes in the ORF marke the three well-conserved catalytic sites. The lengths of each exon (in bp) are presented at the top. The intervening introns are removed for simplicity of illustration, while their phases are marked in parentheses at the corresponding positions. The orthology among introns is indicated by vertical dotted lines. [file 1471-2148-9-72-S5.tiff]
